# Supplementary material for: DNA methyltransferase inhibition induces dynamic gene expression changes in lung CD4+ T cells of neonatal mice with E. coli pneumonia
Source: Sci Rep. 2023 Mar 15;13:4283. doi: 10.1038/s41598-023-31285-5 (PMC10017701; doi:10.1038/s41598-023-31285-5)
Supplement: Supplementary file 1 — Supplementary Legends. [file 41598_2023_31285_MOESM1_ESM.docx]

**Supplemental information**

**Supplemental Figures**

**Supplemental Figure 1**: CD4+ T cell subset marker gene signatures show slight shifts across age groups. **(A)** rlog-normalized expression of selected Th1, Th2, and Treg marker genes, showing that critical markers of all subsets are expressed across age groups and treatment conditions. **(B)** Z-scored expression of markers from (A), highlighting increases in expression for Th1, Th2, and Treg markers in DMSO-treated juvenile mice, corresponding to increased developmental time for thymic maturation. Matrixplots generated using scanpy (Wolf et. al., 2018).

**Supplemental Tables**

**Supplemental Table 1**: Genes differentially methylated in response to DAC treatment across ages.

**Supplemental Tables 2-4:** gProfiler ORA analysis results for genes differentially methylated in (**2**) juveniles, (**3**) neonates, and (**4**) both.

**Supplemental Tables 5-7**: DEseq2 differential expression test results for the (**5**) effect of age (juvenile versus neonatal), (**6**) effect of treatment (DAC versus DMSO), and (**7**) marginal interaction effect between age and treatment.
