# Supplementary figures and images for: DNA methyltransferase inhibition induces dynamic gene expression changes in lung CD4+ T cells of neonatal mice with E. coli pneumonia
Source: Sci Rep. 2023 Mar 15;13:4283. doi: 10.1038/s41598-023-31285-5 (PMC10017701; doi:10.1038/s41598-023-31285-5)

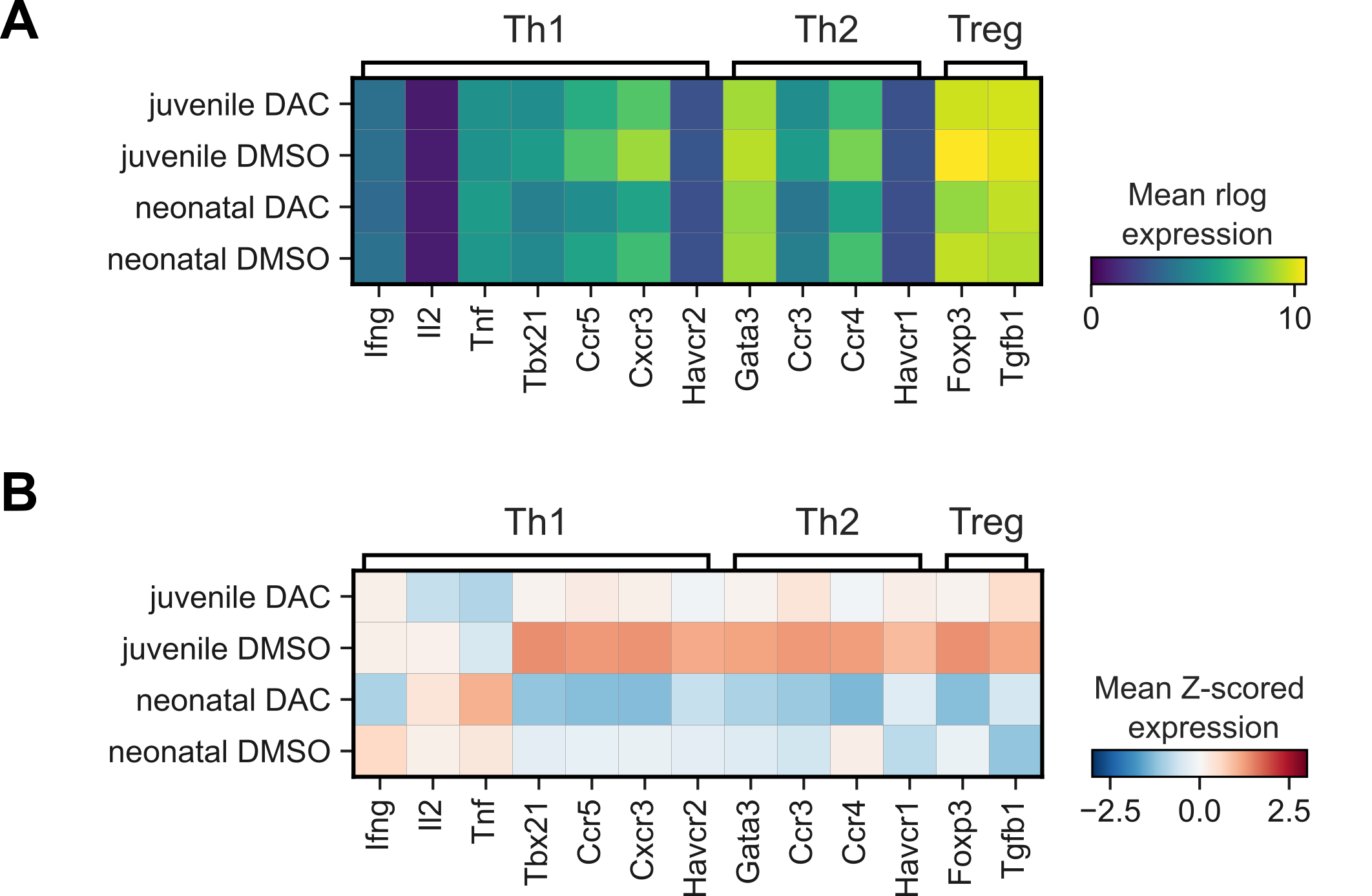

Supplement: Supplementary file 2 — Supplementary Figure S1. [file 41598_2023_31285_MOESM2_ESM.png]
